# Role of Proteomics in Crop Stress Tolerance

**DOI:** 10.3389/fpls.2016.01336

**Published:** 2016-09-08

**Authors:** Parvaiz Ahmad, Arafat A. H. Abdel Latef, Saiema Rasool, Nudrat A. Akram, Muhammad Ashraf, Salih Gucel

**Affiliations:** ^1^Department of Botany, Sri Pratap CollegeSrinagar, India; ^2^Department of Botany and Microbiology, King Saud UniversityRiyadh, Saudi Arabia; ^3^Department of Botany, Faculty of Science, South Valley UniversityQena, Egypt; ^4^Department of Biology, College of Applied Medical Sciences, Taif UniversityTurubah, Saudi Arabia; ^5^Department of Botany, Jamia HamdardNew Delhi, India; ^6^Department of Botany, Government College UniversityFaisalabad, Pakistan; ^7^Pakistan Science FoundationIslamabad, Pakistan; ^8^Centre for Environmental Research, Near East UniversityNicosia, Cyprus

**Keywords:** drought, nutrition, plants, proteomics, salts, temperature

## Abstract

Plants often experience various biotic and abiotic stresses during their life cycle. The abiotic stresses include mainly drought, salt, temperature (low/high), flooding and nutritional deficiency/excess which hamper crop growth and yield to a great extent. In view of a projection 50% of the crop loss is attributable to abiotic stresses. However, abiotic stresses cause a myriad of changes in physiological, molecular and biochemical processes operating in plants. It is now widely reported that several proteins respond to these stresses at pre- and post-transcriptional and translational levels. By knowing the role of these stress inducible proteins, it would be easy to comprehensively expound the processes of stress tolerance in plants. The proteomics study offers a new approach to discover proteins and pathways associated with crop physiological and stress responses. Thus, studying the plants at proteomic levels could help understand the pathways involved in stress tolerance. Furthermore, improving the understanding of the identified key metabolic proteins involved in tolerance can be implemented into biotechnological applications, regarding recombinant/transgenic formation. Additionally, the investigation of identified metabolic processes ultimately supports the development of antistress strategies. In this review, we discussed the role of proteomics in crop stress tolerance. We also discussed different abiotic stresses and their effects on plants, particularly with reference to stress-induced expression of proteins, and how proteomics could act as vital biotechnological tools for improving stress tolerance in plants.

## Introduction

**FIGURE 1:**
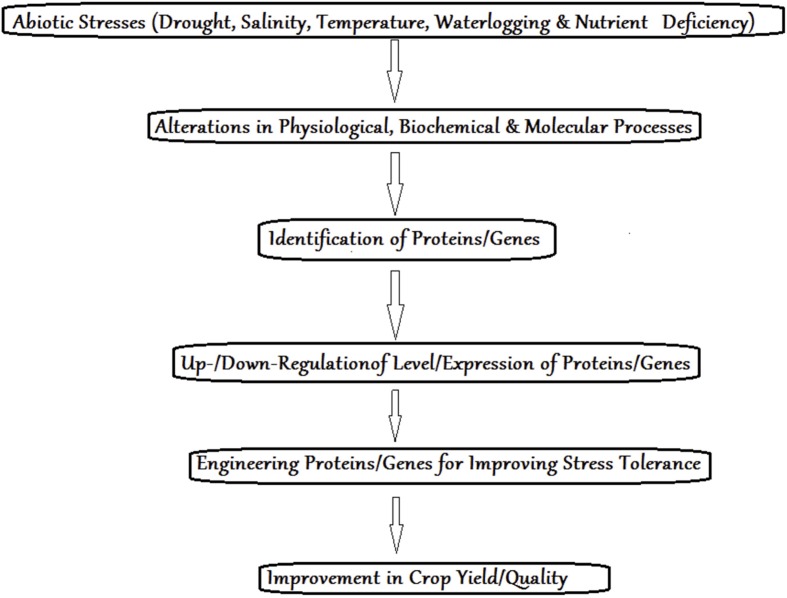
**Improvement in crop yield under stress conditions using proteomic approach**.

As the population of the world increases exponentially, the agriculture sector worldwide is facing a major challenge of ensuring a sufficient food supply to the masses through enhancing agricultural productivity (FAO, 2012). This challenges further intensified with alterations in weather patterns due to changes in climate that impact crop productivity (Ahmad et al., 2012, 2013; Lake et al., 2012). Adverse environmental conditions alter agro-ecological system that may affect the demand for increased agricultural production (Lake et al., 2012; Ahmad et al., 2013). Of various abiotic stresses, drought, salinity, temperature (freezing/heat), light intensity, and heavy metal contamination are the most prevalent that considerably retard not only plant production, but also the quality of crops (Ahmad et al., 2008, 2010, 2012; Ashraf et al., 2011; Ashraf, 2014; Qadir et al., 2014) (**Figure 1**). Plants being sessile in nature during their course of evolution have developed highly sophisticated and effective strategies to counteract environmental cues (Ahmad et al., 2008, 2010, 2012; Haggag et al., 2015). Also they need to adapt quickly to overcome these stresses during their short lifespan. Stress as it is understood today is a factor that alters normal functioning of a number of mechanisms in an organism. During a stress, these mechanisms are up-regulated at various levels of molecular, morphological, and physio-biochemical responses (**Figure 1**). As the stress comes under control, homeostasis is reestablished. In plants under stress conditions, signaling of kinase cascades, ion channels, accumulation of reactive oxygen species (ROS), and hormones are activated (Ahmad et al., 2010, 2012, 2013; Ashraf et al., 2014; Rejeb et al., 2014; Ziogas et al., 2015; Molassiotis et al., 2016).

Recent technological advances have seen the development of the “omics” technologies which are being applied in plant sciences to identify key proteins or metabolites, are novel covering metabolomics, proteomics and, genomics responsible for plants stress tolerance and also the genes regulating such biomolecules (Ahmad et al., 2013; Shelden et al., 2013; Srivastava et al., 2013; Emon, 2016). Application of these omics facilitates a direct observation of the agents affecting plant development. Proteomics deals with determination, identification of proteins, expression profile, post-translational modifications (PTMs), and protein–protein interactions under stress and non-stress conditions (Hashiguchi et al., 2010; Nam et al., 2012; Mertins et al., 2013; Ghosh and Xu, 2014) (**Figure 1**). A notable change in protein expression always takes place in plants under abiotic stresses, so proteomic approach will be very useful in elucidating the role of protein accumulation under stress conditions and its association with stress tolerance (Witzel et al., 2009; Hossain et al., 2012; Perez-Clemente et al., 2013). Proteomic studies in plants under abiotic stresses are well documented, for example: salt stress (Nam et al., 2012; Zhu et al., 2012), drought stress (Castillejo et al., 2008; Caruso et al., 2009; Mirzaei et al., 2012; Mohammadi et al., 2012; Cramer et al., 2013), waterlogging (Komatsu et al., 2009, 2010, 2012, 2013a,b; Alam et al., 2010a,b), and heat stress (Rollins et al., 2013; Xuan et al., 2013) etc.

Proteomics approach is used to investigate the responses of plants to stresses as well as complexity of biochemical processes (Aghaei and Komatsu, 2013; Ghosh and Xu, 2014; Gong et al., 2015). Plant stress proteomics has the ability of identifying possible candidate genes that can be used for the genetic enhancement of plants against stresses (Cushman and Bohnert, 2000; Rodziewicz et al., 2014; Barkla et al., 2016).

Different signaling pathways are reported to be activated in response to stresses resulting in a complex regulatory network involving transcription factors, ion homeostasis, antioxidants, hormones, kinase cascades, ROS, and osmolyte synthesis (Suzuki et al., 2014; Yin et al., 2015). However, the responses of plant cells to abiotic stresses vary in different organs. Organ-specific proteomics combined with subcellular organelle proteomic studies of developmental mechanisms from leaf to root can provide more detailed information for understanding of cellular mechanisms that regulate stress response and signal transduction in various organelles, and they could be used to enhance crop stress tolerance (Komatsu and Hossain, 2013; Yin et al., 2015). Advances in proteomic technologies have widened our genetic and molecular understanding of plant responses under abiotic stresses. However, the main purpose of this review is to present a critical overview of the recent approaches that promise to enhance the tolerance of the plants with a minimum crop loss under different types of abiotic stresses. A detailed study on the modulations or extent of modulations in proteomics of different plants under salinity, water and/or nutrient deficiency, low/high temperature stress as well as under waterlogging conditions have been explained in the present review. Furthermore, how these studies have contributed to crop improvement under abiotic stresses is summarized below.

## Proteomics Approach

### Salinity Tolerance

Within the next 25 years, salinization of arable lands may result in 30% land loss, and up to 50% by the year 2050 (Yan et al., 2003; Wang et al., 2008; Abdel Latef and Chaoxing, 2014; Ma et al., 2015). Thus, improvement of salt tolerance in crops by genetic engineering is contemplated as one of the powerful tools for overcoming the salinity problem world-over (Ashraf and Akram, 2009; Gupta and Huang, 2014).

Plant responses to salt stress through proteomics approach have been studied in both glycophytes and halophytes. Plant biologists have worked with the model plants under saline stress at the proteomic levels, e.g., Jiang et al. (2007) in *Arabidopsis thaliana*, Razavizadeh et al. (2009) in *Nicotiana tobaccum*, Chen et al. (2011) in *Populous cathayana*, Chattopadhyay et al. (2011) in grasspea, and Xu et al. (2011) in *Agrostis stolonifera*. Moreover, agricultural plants have also been investigated under saline stress in different studies, e.g., durum wheat (Peng et al., 2009; Jacoby et al., 2010), canola (Bandehagh et al., 2011), sugarbeet (Wakeel et al., 2011), soybean (Sobhanian et al., 2010), peanut (Jain et al., 2006), *S. bicolor* (Swami et al., 2011; Ngara et al., 2012), maize (Zörb et al., 2009, 2010), tomato (Chen et al., 2009; Manaa et al., 2011), potato (Aghaei et al., 2008), and cucumber (Du et al., 2010) etc.

Apart from glycophytes, halophytes have also been analyzed for the proteomic analysis under salt stress by different workers, e.g., Katz et al. (2007) in *Dunaliella salina*, Wang et al. (2008) in *Physcomitrella patens*, Barkla et al. (2009) in *Mesembryanthemum crystallinum*, Tada and Kashimura (2009) in *Bruguiera gymnorhiza*, Wang et al. (2009) in *Salicornia europaea*, Geissler et al. (2010) in *Aster tripolium*, Pang et al. (2010) in *Thellungiella salsuginea*, Li et al. (2011) in *Suaeda salsa*, and Yu et al. (2011) in *Puccinellia tenuiflora*.

Salt stress can impose its negative effect first on plant roots, because there is an evidence that some salt stress responsive genes and proteins are induced in roots than in shoots (Yan et al., 2003; Hasanuzzaman et al., 2013a). This statement has been authenticated by different workers in soybean (Sobhanian et al., 2010), rice (Liu et al., 2012), wheat (Guo et al., 2012), maize (Zörb et al., 2010), and potato (Aghaei et al., 2008). Proteome of soybean was studied under salt stress by using different tissues (Aghaei et al., 2009; Sobhanian et al., 2010; Ma et al., 2012). They identified 50S ribosome protein which was down-regulated in leaves. This protein is believed to participate in the biosynthesis of soybean protein and causes decrease in plant growth.

Chitteti and Peng (2007) studied changes in the phosphoproteme of roots of rice on exposure to NaCl (150 mM) for a few hours by using Pro-Q Diamond stain. They found 20 proteins upregulated and 18 downregulated. They positively identified 17 of the 20 upregulated proteins and 11 of the 18 downregulated ones. Proteins such as GST, Hsp70, and mannose binding rice lectin up-regulated, while protein kinase, ATP synthase beta-chain, GALP hydrogenase down-regulated. They believed that phosphorylated proteins could be identified using Pro-Q Diamond stain under saline conditions. Of all proteins, 17 overexpressed proteins were responsive to salinity, however, some other proteins identified did not express in any of the proteomic reports on rice on exposure to salinity. All these reports along with some other are listed in **Table 1**.

**Table 1:** Identification and specific roles of different proteins in salt tolerance.

Crop plant	Identified proteins	Role in salt tolerance	Reference
Rice (*Oryza sativa* L.)	APX, DHAR, SOD	Improved leaf sheath and leaf blade	Abbasi and Komatsu, 2004
Pea (*Pisum sativum* L.)	Cu-ZnSOD-II	Superoxide and H_2_O_2_-mediated oxidative damage	Hernandez et al., 1995
Sorghum (*Sorghum bicolor* L.)	Malate dehydrogenase, APX	ROS scavenging	Ngara et al., 2012
Soybean (*Glycine max* L.)	LEA proteins	Seed and hypocotyl development	Aghaei et al., 2009
Potato (*Solanum tuberosum* L.)	Osmotin like protein	Osmotic stress tolerance	Aghaei et al., 2008
Tobacco (*Nicotiana tobaccum* L.)	Osmotin	Osmotic stress tolerance	Abdin et al., 2011
Sugar beet (*Beta vulgaris* L.)	Osmotin like protein	Osmotic stress tolerance	Hajheidari et al., 2005
Sugar beet	Glycine decarboxylase, Ferredoxin-NADP-reductase, Aminomethyltransferase	Membrane bound proteins remained unchanged and resulted in constitutive adaptation at the plasma membrane level	Wakeel et al., 2011
Wheat (*Triticum aestivum* L.)	Glutamine synthase, Glycine dehydrogenase	Improved protein biosynthesis	Caruso et al., 2008
Maize (*Zea mays* L.)	NHX1	Ion transport	Neubert et al., 2005
Barley (*Hordeum vulgare* L.)	*HvNHX1*	Improved salt tolerance due to better ion homeostasis and cell redox homeostasis	Wu et al., 2014
Tobacco	Chitinases and a germin-like protein	Cell wall modifications during plant development remained unchanged	Dani et al., 2005
*Arabidopsis thaliana* L.	STH2, a B-box protein	Positive regulator of photomorphogenesis	Datta et al., 2007
Yeast (*Saccharomyces cerevisiae*)	STO, Salt tolerance protein of *Arabidopsis*	Adversely affect blue light signaling	Indorf et al., 2007


*DHAR, dehydroascorbate reductase; HSPs, heat shock proteins; PRPs, pathogen-related proteins; LEA, late embryogenesis-abundant; NHX1, Na^+^/H^+^antiporter; APX, ascorbate peroxidase; SOD, superoxide dismutase; ABA, abscisic acid; STH2, Salt tolerance homolog 2.*

Following are the major mechanisms that are directly and/or indirectly interlinked with proteins, and proteomics can play a role in regulating them.

#### Photosynthesis

Up-/down-regulation of different proteins predominantly affects photosynthesis by maintaining protein biosynthesis, energy metabolism and detoxification under saline conditions (Rollins et al., 2013; Zhao et al., 2013). Therefore, Parker et al. (2006) attributed the inhibitory effect of NaCl on RuBisCO activase (chaperone protein) which was down-regulated and may be the reason for declined photosynthetic activity in soybean under NaCl stress (Sobhanian et al., 2010). Up-regulation of the 20-kDa chaperonin plays an efficient role in shielding proteins of soybean under salinization (Sobhanian et al., 2010). Furthermore, Zhang et al. (2012) studied the proteomics of 34 different plant species subjected to salinity. They identified 2171 salt responsive proteins and categorized on the basis of cell structure, energy metabolism, CO_2_ assimilation/carbohydrate synthesis, stress and defense interaction, transcription, translation, protein transport and folding, cell division/differentiation and fate and many others with unknown functions.

Recently, Wang et al. (2014) reported 53 differentially expressed protein spots on 2DE maps in *Kandelia candel* subjected to varying salt levels. The results showed the upregulation of proteins involved signal transduction, Na^+^ compartmentalization, photosynthesis, protein folding, and respiration. This protein upregulation was reported to be responsible for enhanced salt tolerance in *K. candel*. Zhu et al. (2012) reported 23 salt responsive proteins in *B. gymnorrhiza* (a halophyte) under salt stress. Ten proteins were reported to be involved in photosynthesis, antioxidative system, protein folding, and cell organization. They also reported different protein expressions at 200 and 500 mM salt stress. At 200 mM salt, over-expression of enzymatic antioxidants and photosynthesis related proteins led to improved plant growth as well as salt tolerance in *B. gymnorrhiza*. *B. gymnorrhiza* is able to sustain severe salt stress due to the upregulation of protein folding and degradation related proteins and cell wall organization related proteins. In salt stressed photosynthetic Chlamydomonas, 3115 proteins were identified, out of which RuBisCO was the most prominent one in the cell (Mastrobuoni et al., 2012).

From the work carried out with different plant species, it is evident that a variety of proteins involved directly/indirectly in the process of photosynthesis are up-/down-regulated under saline conditions. However, it is yet unknown that what types of proteins are specifically involved in all photosynthetic plant species, as protein types and their expression vary from species to species. So, the identification of some specific proteins involved in salinity tolerance and the extent of their expression in different plants could be helpful in improving crop salt tolerance using modern molecular tools.

#### Late-Embryogenesis Abundant (LEA) Proteins

The LEA proteins are synthesized late during embryogenesis in plant seed development. They have also been reported in vegetative plant tissues under different environmental stresses (Xu et al., 1996; Hand et al., 2011; Amara et al., 2012; Battaglia and Covarrubias, 2013). While investigating changes in LEA proteins in rice plants, Chourey et al. (2003) identified four salt-induced LEA proteins which accumulated under salinity, but were degraded when the stress was over. Aghaei et al. (2009) so showed increased expression of LEA proteins under salinity stress in soybean. Xu et al. (1996) introgressed rice plants by HVA1 (a LEA protein gene) isolated from barley. It was found that modified rice plants showed better growth than wild plants under salt stress. Increase in stress tolerance of transgenic rice plants was associated with the high accumulation of the HVA1 protein. Thus, LEA proteins associated genes could be used significantly as a molecular tool for crop improvement under stress conditions.

#### Oxidative Stress/Antioxidants

While studying the role of different types of proteins in rice cultivars/lines with differential stress tolerance Salekdeh et al. (2002) studied the proteome analysis in the roots of two rice cultivars exposed to NaCl (50 and 100 mM). They identified three proteins: caffeoyl-CoA *O*-methyltransferase (CCOMT), involved in lignin biosynthesis, auxin and salicylic acid response (ASR1)-like protein and APX (ascorbate peroxidase). It is worthy to mention that the tolerant rice cultivar had higher amount of ASR1-like protein and CCOMT than in the salt sensitive cultivar, while both cultivars responded almost uniformly to oxidative stress using increased APX. Thus, constitutively expressed proteins improved salt tolerance of the relatively salt tolerant rice cultivar (Vincent and Zivy, 2007)

Witzel et al. (2009) used 2-D gel electrophoresis to compare the root proteomes of two barley cultivars differing in salinity tolerance under salinity stress. Out of total 39 proteins, 26 proteins were isolated using MS (mass spectrometry). In the tolerant cultivar, proteins improved detoxification of ROS due to high accumulation of glutathione. By contrast, in the sensitive one, Fe absorption proteins increased under saline stress.

Yan et al. (2003) found salinity-induced changes in more than 1100 proteins of the proteome of roots in rice. Twelve different proteins were identified, three of these proteins were identified as enolase, four as salt responsive proteins, and the remaining six were new proteins involved in regulating metabolism, nitrogen and carbon in rice for the removal of ROS and the stability of the cytoskeleton (Roveda-Hoyos and Fonseca-Moreno, 2011).

Guo et al. (2013) showed that 17 unique proteins differentially changed in abundance in response to NaCl in *Arabidopsis* roots. These identified proteins were believed to be involved in binding catalysts, signal transduction, transport, metabolism of cell wall and energy, and ROS scavenging and defense.

Overall, very few reports as mentioned under this section are available on protein (s) expression involved in oxidative defense system in plants under salinity stress. Furthermore, it is imperative to mention here that this knowledge is limited only to a very few proteins expressed during saline conditions, whereas not a single report is available on what type of proteins up- or down-regulate under saline conditions or what types of proteins are expressed individually or in combination involved in antioxidative system either enzymatic and/or non-enzymatic.

#### Ion Uptake/Homeostasis

Understanding the proteins particularly root proteins involved in salt response has shown a significant role of these proteins in salt-tolerance mechanisms. Generally, Na^+^ enters the plant roots via apoplastic or symplastic routes, which include many Na^+^ transport transmembrane proteins such as Na^+^/H^+^ antiporters and HKT (a high affinity K^+^ transporter) (Rus et al., 2001; Tester and Davenport, 2013). Different proteins expressed under stress or non-stress conditions are cultivar specific, while some others depend on the level and duration of salinity stress (Peng et al., 2009; Szopinska et al., 2011). For example, Nohzadeh et al. (2007) performed the proteome investigation of plasma membrane of rice cv. IR651 under saline conditions. The 24 different proteins identified were found associated with protein–protein interaction and signaling (reorins and 14-3-3 protein), and proteins involved in controlling K ion channels (Hashiguchi et al., 2010).

Peng et al. (2009) compared the leaf and root proteome of two wheat cultivars under saline stress conditions. They found that majority of the proteins expressed under stress conditions were cultivar specific, while some others were stress responsive. They suggested that improved salinity tolerance in wheat cv. Shanrong No. 3 was associated with more e-flux of toxic byproducts as well as ionic/osmotic homeostasis.

While working with yeast exposed to 0.4 and 1.0 M NaCl for time intervals of 10, 30, and 90 min, Szopinska et al. (2011) identified 88–109 plasma membrane (PM) proteins. Of which, 12 plasma membrane proteins were expressed at mild salt stress (0.4 M) including some already known and some newly target salt-responsive proteins. However, at both salt levels, 20 PM proteins were down-regulated including ABC and/or amino acid transporters, cell wall biogenesis proteins, Pma1, t-SNAREs, and P-type H^+^-ATPase. They found that this protein internalization could be due to alteration in ionic homeostasis or plasma membrane morphology.

Overall, although a number of various types of proteins have been found to be up- and down-regulated in different plant species on exposure to saline regimes, more analyses for identification of different stress responsive proteins and their respective genes involved in different key metabolic pathways is necessary because such proteins could be used in improving stress tolerance in different potential crops using different biotechnological tools.

### Drought Tolerance

Drought is one of the major abiotic constraints that can considerably reduce plant growth and crop yield (Ashraf et al., 2011; Ahmad et al., 2013; Akram and Ashraf, 2013). The reduction in crop yield under drought stress is attributed to water stress-induced osmotic stress, nutritional and hormonal imbalance, activation of oxidative system as well as disturbance in different plant biochemical processes including reduction in carbon uptake through photosynthesis (Hashiguchi et al., 2010; Ashraf et al., 2011; Chugh et al., 2011; Sharma et al., 2012). Shinozaki and Yamaguchi-Shinozaki (2007) reported that there are common genes that are induced during stress in species such as *Arabidopsis* and *Oryza sativa*. Comparative analysis of drought stress using microarrays showed that stress-induced genes of *Arabidopsis* and rice showed a similarity between the two genomes at the molecular level. About 51 genes were identified in *Arabidopsis*, and 73 were reported with similar function in *O. sativa* (Roveda-Hoyos and Fonseca-Moreno, 2011). Larrainzar et al. (2007) studied the proteome of roots of *Glycine max* seedlings under water deficit. They observed a total of 45 proteins under drought stress, only two of them were new proteins. The expression of five proteins was found to be upregulated and that of 21 proteins downregulated. Under the recovery from drought after rewatering soybean plants for 4 days, the concentration of proteins was similar to the control levels. Caruso et al. (2009) studied the proteomics in wheat (*T. aestivum*) under water stress. They detected 36 protein spots out of which 12 proteins were up-regulated and 24 down-regulated. Ke et al. (2009) also studied the proteomics in rice under water deficit conditions using the techniques of 2-DE and mass spectrometry, MALDI-TOF. They detected 18 proteins, and out of these, 12 were up-regulated and 12 down-regulated.

In response to desiccation, Chen et al. (2011) demonstrated the proteomic profiling of seeds of recalcitrant tea. The results showed that 23 proteins up-regulated under desiccation involved in defense against stress as well as redox status under the stress. In another study, wheat plants subjected to drought stress showed 15 bands and out of these 8 protein types were determined to be potential complex forming protein (Zhang et al., 2014). Higher expression levels were found in many proteins of wild genotypes of wheat in response to drought stress. Out of them, 11 protein spots with low peptide matches were identified as candidate unique drought responsive proteins (Budak et al., 2013). Yang et al. (2013) studied protein expression in root of common bean subjected to osmotic stress. They reported 22 proteins differentially regulated by osmotic stress. About 70% of the total expressed proteins were associated with metabolic pathways, such as carbohydrate and amino acid metabolism. Osmotic stress reduced the level of five proteins and increased that of other seven proteins in apoplast (Yang et al., 2013). Sunflower inbred lines (drought tolerant and drought sensitive) were subjected to drought stress and results showed that root metabolism involved proteins that declined in both tolerant and sensitive lines (Ghaffari et al., 2013). The defense related proteins were up-regulated in tolerant lines and down-regulated in sensitive lines (Ghaffari et al., 2013).

Several proteins were found up/down-regulated in different plant species as mentioned in above reports on exposure to drought stress. Some of the important mechanisms perturbed or therein proteins involved for playing their roles in improving drought tolerance in different plant species are listed below such as:

#### Photosynthesis

It is well known that the efficiency of photosynthesisdepends on number and activity of proteins (Deeba et al., 2012; Galvan et al., 2013). A study carried-out by de Almeida et al. (2013) on two varieties of sugarcane RB 72910 (drought tolerant) and RB 943365 (drought sensitive) showed change in protein expression. The expression of some proteins in RB 72910 was up-regulated and that in some others down-regulated. However, in cv. RB 943365 all the proteins showed down-regulation. These proteins were associated with functions such as photosynthesis, signal transduction and regulation processes. Valero-Galván et al. (2013) showed a decrease in protein abundance/expression mainly those involved in ATP synthesis and photosynthesis upon water withholding/water deficiency in holm oak (*Quercus ilex*).

The down-regulation of glycolytic enzymes under osmotic stress may be a strategy for accumulating sugars as an energy source for attaining enhanced growth after recovery of drought stress. Hajheidari et al. (2005) while working with sugar beet leaves identified different proteins involved in redox regulation, photosynthesis (Rubisco), oxidative defense system, and chaperone treated with drought. Recently, Gil-Quintana et al. (2013) have shown that on exposure to drought stress, cell growth of soybean plants reduced due to reduction of many proteins having a role in amino acid metabolism, carbon metabolism, as well as protein synthesis. In an earlier study, Aranjuelo et al. (2011) while carrying out proteomic analysis of alfalfa plants have shown that decreased carboxylation activity due to water shortage was associated with decrease in Rubisco protein content, its activation as well as regeneration. Furthermore, drought-induced reduction in amino acids (glutamic acid and asparagine) showed that N availability was also limited due to decline in nitrogenase activity while increase in that of proteases. In cotton plants, although Deeba et al. (2012) examined that 16 protein spots were up-regulated while 6 down-regulated, however, an additional information on the molecular basis of drought intolerance in cotton plants still needs to be determined. Rollins et al. (2013) determined drought-induced changes in the barley leaf proteome using mass spectrometry and differential GE. They showed that although water stress induced a substantial decrease in plant biomass and yield production of barley, photosynthetic efficiency as well as proteomics remained unchanged due to drought stress.

From the above-mentioned all reports it can be concluded that plant photosynthetic efficiency was adversely affected by drought-induced decrease in activities/levels of different proteins, particularly those of rubisco. Furthermore, many other proteins were also visualized that were markedly affected by drought stress, but their characterization is still underway.

### Late-Embryogenesis Abundant (LEA) Proteins

Late-embryogenesis abundant proteins are water soluble proteins that are synthesized in high concentration in desiccation-tolerant plant (Alam et al., 2010a,b). The accumulation of dehydrin and ferritin were identified in proteomic investigation of soybean roots under drought stress (Alam et al., 2010a,b; Nouri et al., 2011). Dehydrins are LEA proteins, can effectively improved plant growth under stress by reducing the harmful effect of ROS (Grelet et al., 2005; Nouri et al., 2011; Hossain et al., 2013). Grelet et al. (2005) identified a LEA protein, PsLEAm localized within the matrix space of seed mitochondria of *Pisum sativum*. PsLEAm shows characteristic of LEA proteins and usually expresses during late seed development. It could not be detected in vegetative tissues, but on exposure to severe water stress, it expresses in leaves. The authors suggested that under drought stress conditions in an *in vitro* assay, it can play a beneficial role during desiccation. In *Medicago truncatula* seeds, about 15 polypeptides were reported to be expressed under drought stress, which were significantly associated with drought tolerance of the crop. Of all, 11 polypeptides were identified as LEA proteins including MtEm6, isoform of dehydrins, MtPM25, MP2, PM18, and all isoforms of SBP65. Alteration in the abundance of MtEm6 and MtPM25 in imbibed *M. trunculata* seeds during the loss of drought tolerance and in developing embryos during the acquisition of drought tolerance confirmed the involvement of these two proteins in drought tolerance (Boudet et al., 2006). Functional diversity among LEA proteins was confirmed in maize by Amara et al. (2012). Protein visualization showed that cells expressing LEA protein Mg3–GFP, were better in controlling cell shrinkage. Another potential mitochondrial LEA protein LEAM was found to be expressed in seeds and this was reversibly folded into α-helices upon water shortage. Generally, this LEA protein protects liposomes by interacting with membranes under water deficit conditions which then protects the inner mitochondrial membrane under desiccation.

Although a variety of LEA proteins have been found to be overexpressed in different plant species under drought stress and have shown their potential role in drought tolerance, the mechanisms of protection from drought are still being researched.

#### Oxidative Stress/Antioxidants

Plants accumulate antioxidants to counteract stress-induced ROS. For example, upregulation of superoxide dismutase (SOD), an ROS scavenger, was reported in soybean (Toorchi et al., 2009) and rice (Ali and Komatsu, 2006) under drought stress. The study of Caruso et al. (2009) identified 21 different proteins including some isoforms and subunits of enzymes under water stress. They reported that 18% of the identified proteins were associated with the routes of glycolysis and gluconeogenesis, 15% proteins were associated with the removal of ROS, 12% in biosynthesis of amino acids, 9% in the Calvin cycle, 6% with defense mechanisms, and the remaining 3% related to post-transcriptional regulation. Benešová et al. (2012) have shown that drought stress induced up-regulation of stress-related protective proteins namely chaperones and dehydrins in two differential tolerant cultivars of maize. The alteration in the concentrations of different detoxification proteins significantly associated with the enzymatic antioxidants, generally lower in the sensitive maize cultivar due to reduced level of proteosynthesis and changes in the translation machinery. In grapevine (*Vitis vinifera* L.) Cramer et al. (2013) have reported that proteomic responses to water stress generally involved abundance of proteins for translation, as well as steroid and antioxidants metabolism. Recently, Das et al. (2016) investigated the effect of heat and drought alone or combine in two soybean varieties, Surge and Davison using 2D-DIGE proteomic technique. They found that photosynthesis-related proteins affect RuBisCO regulation, electron transport, Calvin cycle, and carbon fixation under these stresses. In addition, carbonic anhydrase accumulation in the cell helps the cell to become more resistant to cytotoxic concentrations of hydrogen peroxide. While working with sunflower proteomics, Ghaffari et al. (2013) examined that on exposure to drought stress, defense/disease and energy involved proteins reduced significantly in the relatively less tolerant sunflower cultivar, while they increased in the tolerant one. They suggested that better water transport, energy usage, and antioxidant defensive system are essential mechanisms for regulating plant growth under water limited environment. In drought-stressed creeping bentgrass (*A. stolonifera* L.) plants, fifty-six stress-responsive proteins visualized, of which some proteins those were participated in C and N metabolisms were suppressed due to drought stress. However, glutathione-*S*-transferase, APX and CAT (antioxidant enzyme) proteins were up-regulated in a relatively drought tolerant cv. Penn-A4, which suggests that proteins have an effective role in drought tolerance by maintaining cell turgor, membrane stability, cell wall expansion and regulation of ROS defensive system under drought stress (Xu and Huang, 2012). It has been identified by Tanou et al. (2012) that local and systemicH_2_O_2_-oxidative and NO-nitrosative bursts involved in encoding proteins associated with H_2_O_2_ production such as NOX, Fe SOD, Cu/Zn SOD, and Mn SOD as well as NO biosynthesis (e.g., NOS, NiR, and NR) after 8 days of salinity stress. Recently, Yin et al. (2015) reported that exogenous application of calcium increased the salinity tolerance of soybeanseedlings by promoting protein biosynthesis, inhibiting proteolysis, redistributing storage proteins, regulating protein processing in endoplasmic reticulum, and enriching antioxidant enzymes and activating their activities. Several signaling pathways activated in response to multiple stresses have been revealed in transcriptome, metabolome, and proteome analyses of different plants subjected to different stresses, resulting in a complex regulatory network involving antioxidants, hormones, transcription factors, kinase cascades, ROS, and osmolyte synthesis (Suzuki et al., 2014; Yin et al., 2015). However, differentially adapted species vary in their response to stressinduced oxidative stress. A reasonable number of reports are available in the literature on drought-induced increase or decrease in the levels of a number of enzymatic and non-enzymatic antioxidants. Mostly an increase in ROS served as a signal, which triggered a biochemical response to establish the redox balance of the cell. Identification of a number of PTMs is an important feature of the proteomic approach in response to oxidative stress involves which have been dicussed but have rarely been the focus of studies investigating the response to environmental stress. The implification of proteomics for the functional analysis of plants will benefit from advances in plant phenotyping particularly automated, non-invasive phenotyping of plant collections will assist in characterizing the relevant traits for future crop breeding.

#### Abscisic Acid (ABA) Metabolism

Abscisic acid (ABA) plays a major role in plant response to drought stress as it controls the closing of stomata to minimize water loss. Previously, Zhao et al. (2008) studied a guard cell proteome under drought stress in *A. thaliana*. They detected 336 new proteins (not detected before in guard cell) with fifty-two proteins involved in signal transduction. Of these proteins, the myrosinase TGG1 was associated with ABA metabolism and stomatal regulation (Hashiguchi et al., 2010). Also, Nishikawa et al. (2008) stated that the modification of ABA signaling plays a role in drought tolerance of plants. In their work on proteome analysis of *Arabidopsis* under drought stress, they found that the improvement of fresh weight in *Arabidopsis* under water stress was through suppression of water vapor loss from stomata. This vapor loss was associated with the high level of sphingosine-1-phosphate. It is worthy to mention that, ABA controlled the sphingosine-1-phosphate level through sphingosine kinase (Hashiguchi et al., 2010)

Recently, Alvarez et al. (2014) have observed significantly more expression of proteins in two differential wheat cultivars, Nesser and Opata. They observed a comparatively higher number of ABA-responsive proteins in the roots of wheat cv. Nesser as compared to those in cv. Opata, which confirmed the role of these ABA responsive proteins in enhancing drought tolerance. ABA has a well known protective role in stomatal closure under water limited environment, but very rare information is available in the literature on proteomic analyses relevant to ABA accumulation. In addition, how and what type of proteins are up- or down-regulated and their association with drought-induced increase in ABA still needs to be elucidated.

Overall, a number of reports are available in the literature on identification of different proteins in water stressed plants, but little information is available on their regulation and identification. Thus, there is a need to examine the regulation of all proteins being identified in different plant species exposed to water stress. In addition, the actual function of stress responsive proteins is not fully known which needs to explored with particular reference to plant tolerance mechanisms.

### Temperature Tolerance

#### High Temperature Stress

High temperature stress (heat stress) results in disturbance in cellular homeostasis and can cause drastic reduction in growth, development and even death of plants (Hasanuzzaman et al., 2013b; Brosché et al., 2014). High temperature induces the synthesis of high (60–110 kDa) and small (15–45 kDa) molecular mass HSPs in plants (Miernyk, 1997; Renaut et al., 2006). Lee et al. (2007) studied the proteomics analysis in rice leaves under heat stress. They identified 48 proteins on exposure to 12 to 24 h of high temperature versus control. Out of all identified proteins, 18 were HSPs including smHSPs, HSP100, HSP70, dnak-type molecular chaperone BiP and Cpn60. Zhu et al. (2006) reported that the induction of HSP70 in transgenic soybean plants by introgressing HsfA1 enhanced tolerance to high temperature stress. Skylas et al. (2002) detected seven HSPs spots in relatively heat tolerant wheat cv. Fang as compared to heat sensitive wheat cv. Wyuna. High accumulation of HSPs in plants is generally associated with heat tolerance (Hashiguchi et al., 2010; Xu et al., 2011; Hasanuzzaman et al., 2013b). HSPs could be categorized into five different sub-groups depending on their molecular weight (Kosova et al., 2011).

Süle et al. (2004) identified *S*-adenosylmethioninesynthetase (proteins other than HSPs) as a tolerance marker in heat-tolerant and susceptible barley cultivars. In other studies, a high accumulation of eIF4F and eIF5A-3 (eukaryotic translation initiation factors) has been reported to induce cellular reorganization leading to PCD on a long-term exposure to high temperature (Zhang et al., 2010; Liu et al., 2013; Rollins et al., 2013).

Rollins et al. (2013) reported 99 proteins expressed differentially in barley under heat stress. These regulated proteins were associated with energy metabolism, photosynthesis, detoxification, and translation. Liu et al. (2013) studied leaf proteome in two cvs. King (heat sensitive) and Vista (heat tolerant) of *S. splendens*. The results revealed 1213 leaf proteome spots of large size. Of which, 33 proteins were differentially expressed when *S. splendens* plants were subjected to high temperature stress. These proteins regulate photosynthesis and protein processing under heat stress. Li et al. (2013) reported 81 over-expressed proteins involved in protein synthesis, storage, transport, in/out-flux, signal transduction as well as defensive system against diseases in alfalfa subjected to heat stress. The proteome study of Pinelliaternata leaves subjected to high temperature stress showed 600 protein spots, 7 of which down-regulated and 20 up-regulated (Zhu et al., 2013). Of the 24 proteins identified, maximum of them were sHSPs associated with chlorophyll biosynthesis, RNA processing, photosynthesis as well as protein denaturation/degradation (Zhu et al., 2013). Liao et al. (2014) studied the proteomics at early milky stage of rice grains after exposure to heat stress. The results of 2-DE revealed about 27 up-regulated proteins in rice grains, predominantly from the heat tolerant lines. Out of total 27 proteins, 25 differentially expressed proteins are involved in biosynthesis, energy metabolism, oxidation, heat shock metabolism, and regulation of transcription.

Under high temperature stress, majority of the proteins expressed fall under the heat shock proteins (small and large molecular weight), and their expression varies between heat stress tolerant and sensitive cultivars. Identification and introgression of heat stress tolerant proteins could be used in modern biotechnology tools for the improvement of stress tolerance in economically important crops all-over the world.

#### Low Temperature Stress

Temperature is a main environmental factor which affects growth, productivity and distribution of plants (Shah et al., 2011; Bita and Gerats, 2013; Zia et al., 2014; Zinn et al., 2014). The phenomenon of exposing plants to temperatures from 0 to 15°C (non-freezing temperatures) is called cold stress or chilling stress (Renaut et al., 2004). Cold stress is associated with reduction of water absorption resulting in cellular desiccation (Sinclair et al., 2013). Also cold stress induces alteration in metabolites leading to an oxidative stres (Renaut et al., 2004; Kosova et al., 2011; Sinclair et al., 2013). The increased freezing tolerance by plants under low temperature is called cold acclimation (Renaut et al., 2004; Timperio et al., 2008; Kosova et al., 2011; Miura and Furumoto, 2013). It includes different changes in protein and gene expression as well as metabolites (Renaut et al., 2006; Miura and Furumoto, 2013).

Kawamura and Uemura (2003) studied plasma membrane proteome of *Arabidopsis* under low temperature. They detected 38 proteins, 27 of which were soluble, whereas 15 insoluble. Imin et al. (2004) reported 70 proteins including 12 new (47 up- and 11 down-regulated) at pH between 4.0 and 7.0 in rice anthers exposed to cold stress (12°C) for 48 h. A proteomic work on mitochondria of pea (*Pisum sativum*) reported by Taylor et al. (2005). Twenty, out of 33 proteins appearing in response to cold stress at 4°C for 1.5 day. Cheng et al. (2009) in their study on proteomics of soybean seeds subjected to cold stress (4°C), reported 40 proteins (25 up- and 15 down-regulated). These proteins are involved in cell growth/division, storage, defense of cell, energy protein synthesis, transcription, and transport. Toorchi et al. (2009) studied proteomics analysis of soybean seedlings under cold stress for 2 days and observed overexpression of pathogenesis-related protein 1 (PR1), while down-regulation of caffeoyl-CoA 3-*O***-**methyltransferase and PR10 proteins. Komatsu et al. (2009) identified a total of 12 *N*-glycosylated proteins in rice sheaths under cold stress. Of them, a calreticulin protein controlled phosphorylation and glycosylation in leaf sheathes under low temperature stress, indicating that calreticulin may regulate the expression of several other proteins (Hashiguchi et al., 2010).

LB-a is one of the freezing stress responsive low abundant proteins and identified as Hsp70 which decreased in rice plants exposed to low temperature stress (Hashimoto and Komatsu, 2007; Bashir et al., 2010). This decrease in LB-a might be attributed to cold-induced chloroplast degradation (Bashir et al., 2010). The proteomic analysis of plants under low temperature indicated an increase in enzymes involved in ROS scavenging, e.g., Imin et al. (2004) reported high accumulation of different isoforms of APX in tri-nucleate rice pollen under cold stress. Degand et al. (2009) showed that Cu/Zn-SOD abundantly increased in chicory roots under cold stress. While Kosmala et al. (2009) detected an enhancement in enzymes which take part in AsA and GSH metabolism in Festucapratensis plants. Many authors reported the accumulation of chaperonins (chaperonins 60 and 20) and HSPs (HSP90, HSP70) under low temperature stress (Kawamura and Uemura, 2003; Taylor et al., 2005).

Freezing injury is a result of drastic low temperature conditions (Perez-Munuera et al., 2009). Freezing temperature induces desiccation, and imbalances plasma membranes leading to formation of inverted hexagonal phase membrane structure (Sung et al., 2003; Timperio et al., 2008). Timperio et al. (2008) reported that anti-freezing proteins (AFPs) play a significant role in maintaining plant growth against freezing injury. AFPs are similar to the pathogenesis-related protein involved in eliminating freezing stress and inducing disease resistance (Timperio et al., 2008). Winter cereal such as winter rye and wheat accumulated AFPS in their apoplast to tolerate freezing stress (Marentes et al., 1993).

Xu et al. (2012) reported a significant increase in relative abundance of antioxidant related proteins in low temperature tolerant wheat cv. Shixin 828 compared to those in sensitive cv. Shiluan 02-1. They also reported that carbohydrate metabolism related proteins were more abundant in cv. Shiluan 02-1. Xuan et al. (2013) studied the proteomics of *Z. japonica* (cold tolerant cv. Meyer) and *Z. metrella* (cold sensitive cv. Diomond) under cold stress. They showed that 700 proteins were resolved on 2-DE gels, but only 70 protein were considerably over-expressed. They suggested that of all identified proteins, 45 proteins were participated in cellular metabolic processes. Cultivar Meyer showed considerably high concentration/number of accumulated proteins as compared to cv. Diamond and only cv. Meyer showed 15 increased proteins under cold stress. The cold responsive proteins have been associated with the biosynthesis of carbohydrates, proteins, and nucleotides, ROS scavenging, proteolysis, protein folding, and energy storage.

## Conclusions and Future Prospects

Abiotic stresses like salinity, drought, high temperature, freezing stress, water-logging, and mineral toxicity and deficiency severely affect crop productivity and such losses are of major concern for all nations so as to cope with the increasing food demand. Abiotic stresses are known to hinder plant growth and yield by causing a variety of adverse effects including disturbance in regulation of many proteins involved in protein folding, ROS scavenging, proteolysis, metabolic energy supply, biosynthesis of carbohydrates and nucleotides, signal transduction, PCD, RNA processing, redox homeostasis, energy metabolism, secondary metabolites, glycolysis, lipid peroxidation, ethylene biosynthesis and cell wall loosening, etc. However, regulation of different proteins varies among species, therefore complete dissection of all proteins involved in different metabolic processes plant species under a variety of stresses needs to be further carried out.

The vital physiological process, photosynthesis that distinguishes green plants from other organisms entirely depends on the photosynthetic machinery along with the activities/levels of different proteins including rubisco. Furthermore, many other proteins have been also visualized that are markedly affected by stress, but their complete characterization is still underway.

Stress-induced increase or decrease in the levels of a number of enzymatic and non-enzymatic antioxidants in plants is now widely reported, but what type of a particular protein is produced during oxidative stress and what types of proteins are involved to control its accumulation at different levels of stress remains unclear. Furthermore, it is imperative to mention here that this knowledge is limited only to a very few proteins expressed during water stress and saline conditions, whereas not a single report is available on what type of proteins up- or down-regulate under different stress conditions or what types of proteins are expressed individually or in combination involved in antioxidative system.

Nutrients (macro/micro) are effectively involved in regulation of plant metabolism. Deficiency of anyone of these triggers a number of pathways, more promising of which are carbohydrate metabolism, protein homeostasis, antioxidative defenses, signal transduction, membrane transductions, etc. Thus, it seems plausible to identify proteins which express under nutrient deficient environments, so they could be considered as potential indicator of mineral deficiency in plants. Furthermore, extensive research has been carried out for the determination of levels, deficiency symptoms, modes of action, and QTLs of different nutrients, but very few reports are available on the introgression of nutrient-related genes or/and QTLs to overcome nutrient deficiency within plant cells/tissues. So, research to explore this knowledge would be more beneficial for improving tolerance to nutrient deficiencies in crop plants.

Under high temperature stress, majority of the proteins expressed fall under the heat shock and LEA proteins. Their expression varies between heat stress tolerant and sensitive cultivars. Thus, identification and introgression of proteins expressed in heat stress tolerant plants could be used in modern biotechnology tools for the improvement of stress tolerance in economically important crops all-over the world.

Proteomic approach has been found to be very important as it helps plant physiologists to understand what is going on in the cell due to an external stimulus. Proteomics has gained attention world-wide due to easy handling of the proteomic analysis tools and accuracy of the results. For example, a number of techniques have been employed for the separation and identification/characterization of different proteins in different plant species including 2-dimensional liquid chromatography (2D-LC), polyacrylamide gel electrophoresis (PAGE), sodium dodecyl sulfate (SDS)-PAGE, pro-Q Diamond stain, 2-D gel electrophoresis, mass spectrometry, Coomassie brilliant blue (CBB)-stained 2-DE, MALDI-TOF, fluorescence, 2-D PAGE, non-gel-based LC-MS, ion-exchange chromatography (IEC), and 2-D difference GE (2D-DIGE). All these techniques have yielded sound results on characterization of proteins.

## Author Contributions

PA, AA, and SR wrote the manuscript. NA, MA, and SG contributed in section 2 of this manuscript. They also reviewed and updated the manuscript.

## Conflict of Interest Statement

The authors declare that the research was conducted in the absence of any commercial or financial relationships that could be construed as a potential conflict of interest.
